# CODEHOP-mediated PCR – A powerful technique for the identification and characterization of viral genomes

**DOI:** 10.1186/1743-422X-2-20

**Published:** 2005-03-15

**Authors:** Timothy M Rose

**Affiliations:** 1 Department of Pathobiology, Box 357238, School of Public Health and Community Medicine, University of Washington, Seattle, WA 98195, USA

## Abstract

Consensus-Degenerate Hybrid Oligonucleotide Primer (CODEHOP) PCR primers derived from amino acid sequence motifs which are highly conserved between members of a protein family have proven to be highly effective in the identification and characterization of distantly related family members. Here, the use of the CODEHOP strategy to identify novel viruses and obtain sequence information for phylogenetic characterization, gene structure determination and genome analysis is reviewed. While this review describes techniques for the identification of members of the herpesvirus family of DNA viruses, the same methodology and approach is applicable to other virus families.

## Introduction

Only a very small fraction of the vast number of viral species belonging to the different virus families have been identified and characterized to date. The majority of these uncharacterized viral species are found in host organisms which have not been targeted in biomedical, plant or animal research. However, recent reports have noted an increase in the occurrence of viral diseases, not only in humans, but in animals and plants as well. While some of this rise may reflect more effective surveillance techniques, disease outbreaks caused by novel cross-species infections and/or subsequent virus recombination events have occurred [1]. Therefore, the development of tools for the detection of viruses, the characterization of their genomes and the study of their evolution, becomes important, not only for basic scientific study, but also for the protection of public health and the well-being of the plant and animal life that surrounds us.

We have developed a novel technology to identify and characterize distantly related gene sequences based on consensus-degenerate hybrid oligonucleotide primers (CODEHOPs)[2]. CODEHOPs are designed from amino acid sequence motifs that are highly conserved within members of a gene family, and are used in PCR amplification to identify unknown related family members. We have developed and implemented a computer program that is accessible over the World Wide Web to facilitate the design of CODEHOPs from a set of related protein sequences [3]. This site is linked to the Block Maker multiple sequence alignment site [4] on the BLOCKS WWW server [5] hosted at the Fred Hutchinson Cancer Research Center, Seattle, WA.

We have utilized the CODEHOP technique to develop novel assays to detect previously unknown viral species by targeting sequence motifs within stable housekeeping genes that are evolutionarily conserved between different members of virus families. Using CODEHOPs derived from conserved motifs within retroviral reverse transcriptases, we have previously identifed a diverse family of retroviral elements in the human genome [2], as well as a novel endogenous pig retrovirus [6], and a new retrovirus in Talapoin monkeys [7]. We have also developed assays to detect unknown herpesviruses by targeting conserved motifs within herpesvirus DNA polymerases. Using this approach, we have identified fourteen previously unknown DNA polymerase sequences from members of the alpha, beta and gamma subfamilies of herpesviruses [8], and have discovered three homologs of the Kaposi's sarcoma-associated herpesvirus in macaques [9,10]. We have also used the CODEHOP technique to clone and characterize the entire DNA polymerase gene from these new viruses [10] and to obtain sequences for larger regions of viral genomes containing multiple genes, targeting the divergent locus B of macaque rhadinoviruses [11]. The sequence information obtained from the amplified gene and genomic fragments from these studies has allowed informative phylogenetic characterization of the new viral species, and has provided critical information regarding the gene structure and genetic content of these unknown viral genomes.

In this review, the CODEHOP methodology and its utilization in the identification and characterization of novel viral genomes using the herpesvirus family as an example is described. Published CODEHOP assays that we have previously used to identify new herpesviruses are discussed and the latest refined assays and their utility are provided. The use of the CODEHOP methodology for the analysis of larger regions of viral genomes is presented along with the general application of this technology for the identification of viral species and their genes in other virus families. Finally, the software and Web site that we have developed to derive CODEHOP PCR primers from blocks of multiply aligned protein sequences are described.

## CODEHOP Methodology

### General CODEHOP Design and PCR Strategy

**Figure 1:**
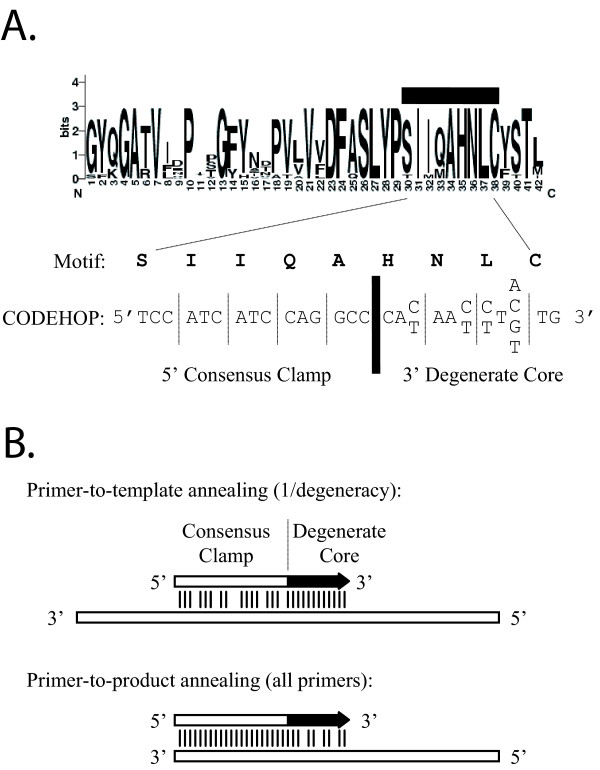
**CODEHOP description and PCR strategy. **(A) A conserved DNA polymerase sequence motif in LOGOS representation [31] and a sense-strand CODEHOP (HNLCA) derived from that motif is shown. The 3' degenerate core contains all possible codons encoding four conserved amino acids and has a degeneracy of 32. The 5' clamp contains a consensus sequence derived from the most frequently used codons for 5 upstream amino acids within the motif. (B) Schematic description of the CODEHOP PCR strategy illustrating regions of mismatch in primer-to-template annealing during the early PCR cycles and primer-to-product annealing during subsequent cycles. Vertical lines indicate matches between primer (arrow) and template or amplified PCR product. The overall degeneracy of the 3' degenerate core is the product of the degeneracies at each nucleotide position so that the fraction of primers with sequences identical to the targeted template across the degenerate core = 1/degeneracy.

CODEHOPs are derived from highly conserved amino acid sequence motifs present in multiple alignments of related proteins from a targeted gene family. Each CODEHOP consists of a pool of primers where each primer contains one of the possible coding sequences across a 3–4 amino acid motif at the 3' end (degenerate core) (Figure 1A) [2]. Each primer also contains a longer sequence derived from a consensus of the possible coding sequences 5' to the core motif (consensus clamp). Thus, each primer has a different 3' sequence coding for the amino acid motif and the same 5' consensus sequence. Hybridization of the 3' degenerate core with the target DNA template is stabilized by the 5' consensus clamp during the initial PCR amplification reaction (Figure 1B). Hybridization of primers to PCR products during subsequent amplification cycles is driven by interactions through the 5' consensus clamp.

Conserved amino acid motifs used for CODEHOP design are identified by alignment of related proteins from a targeted gene family using computer programs such as the Clustal W multiple alignment program [12]. Optimal blocks contain 3–4 highly conserved amino acids with restricted codon multiplicity from which the 3' degenerate core is derived; the presence of serines, arginines and leucines are not favored due to the presence of six possible codons for each amino acid. In addition, optimal blocks contain 5 or more conserved amino acids from which the 5' consensus clamp is derived. These blocks of conserved amino acid sequences should be situated in close enough proximity to allow efficient PCR amplification between blocks yet distant enough to flank a region of significant sequence information.

We have developed web-based software to predict CODEHOP PCR primers from blocks of conserved amino acid sequences [2,13]. Multiple related protein sequences from the targeted gene family are provided to the Block Maker program [4] at the BLOCKs WWW server [5] which produces a set of conserved sequence blocks obtained from a multiple sequence alignment. The sequence block output is linked directly to the CODEHOP design software [3] which predicts and scores possible CODEHOP PCR primers. The different CODEHOP PCR primers discussed in this review were either designed manually or with the CODEHOP software, and are listed in Table 1.

**Table 1:** CODEHOPs developed for herpesvirus screens targeting the DNA polymerase

CODEHOPS (degeneracy)^1^	Bias^2^		Sense	5'>3' Sequence(degenerate codons are in lower case)^3^
			
	3' Core	5' Clamp		
**"TVG-IYG" Assay**^4^				
DFA (512)	All HV (IHV, HHV6,7)	NA^5^	+	Gayttygcnagyytntaycc
ILK (1024)	All HV		+	TCCTGGACAAGCAGcarnysgcnmtnaa
TGV (256)	All HV (IHV, HHV6,7)	-^6^	+	TGTAACTCGGTGtayggnttyacnggngt
IYG (48)	All HV (IHV, AlHV1, RRV)	-	-	CACAGAGTCCGTrtcnccrtadat
KG1 (128)	All HV	-	-	GTCTTGCTCACCAGntcnacnccytt
**"DFASA-GDTD1B" Assay**^7^				
DFASA (256)	All HV (IHV, HHV6,7)	-	+	GTGTTCGACttygcnagyytntaycc
VYGA (256)	All HV (IHV)	-	+	ACGTGCAACGCGGTGtayggnktnacngg
GDTD1B (64)	All HV	-	-	CGGCATGCGACAAACACGGAGTCngtrtcnccrta
**"QAHNA" Assay**^7^				
QAHNA (48)	αHV γHV (IHV, βHV)	(CMV)	+	CCAAGTATCathcargcncayaa
**"SLYP" Assay**^8^				
SLYP1A (64)	All HV (CMV, EHV2)	-	+	TTTGACTTTGCCAGCCTGtayccnagyatnat
SLYP2A (128)	CMV (All other HV)	-	+	TTTGACTTTGCCAGCCTGtayccntcnatnat
**CODEHOP Predicted**^9^				
HNLCA (32)	All HV (IHV)	CODEHOP^10^	+	TCCATCATCCAGGCCcayaayytntg
VYG1A (128)	All HV (IHV)	CODEHOP	+	GCAACGCGGTGTACggnktnacngg
YGDTB (16)	All HV	CODEHOP^11^	-	CGGCATGCCATGAACATGGAGTCCGTrtcnccrta
KGVDB (32)	All HV	CODEHOP	-	CTTCCGCACCAGGTCnacnccytt

^1 ^The degree of degeneracy, ie the number of individual primers in the pool, is given in parentheses.

^2 ^Bias indicates the reliance on a specified subset of sequences for determination of the 3' degenerate core or 5' consensus clamp. Sequences which are biased against by the choice of nucleotide sequences are indicated in parentheses (see the multiple sequence alignments from which the primers were derived in Figures 3-6).

^3 ^IUB code: Y = T, C; R = A, G; K = G, T; M = A, C; H = A, C, T not G; N = A, C, G, T.

^4 ^[8]

^5 ^NA, not applicable

^6 ^(-), no specific design bias

^7 ^[9]

^8 ^Primers predicted manually.

^9 ^Primers predicted using the CODEHOP software.

^10 ^Clamp sequence was predicted by the CODEHOP software using default codon usage table and thus had no inherent bias design

^11^Underlined sequences have been added to the primer predicted by the CODEHOP software (see legend to Figure 4) Abbreviations: HV, herpesvirus; αHV, alphaherpesvirus; βHV, betaherpesvirus; γHV, gammaherpesvirus; AhlHV1, alcelaphine herpesvirus 1; CMV, cytomegalovirus; EHV2, equine herpesvirus-2, HHV6, human herpesvirus 6; HHV7, human herpesvirus 7; IHV, ictalurid herpesvirus (catfish)

### CODEHOP PCR Amplification, Product Cloning and Sequence Analysis

CODEHOP PCR amplification has been performed using classical and touch-down approaches with a hot-start initiation [2]. More recently, thermal gradient PCR amplification has been used to empirically determine optimal annealing and amplification conditions for the pool of primers [11]. Different buffers, salt concentrations, and enzymes have been employed with varying success due to differences in DNA template preparation and the unknown nature of the targeted sequence. PCR products are either sequenced directly or after TA-cloning.

In this review, sequences were compared by BLAST analysis [14] and multiple alignment using Clustal W [12]. Phylogenetic analysis of the multiply aligned sequences was performed using protein distance and neighbor-joining analysis implemented in the Phylip analysis package [15]. Bootstrap analysis was also performed with 100 replicates and a consensus phylogenetic tree was determined. For the phylogenetic analysis, positions in the multiple alignment containing gaps due to insertions or deletions within the sequence blocks were eliminated.

## The "TGV-IYG" CODEHOP assay to detect novel herpesviruses

**Figure 2:**
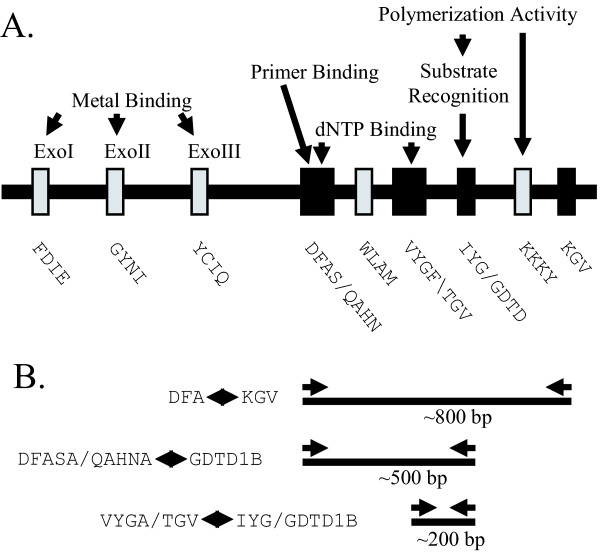
**CODEHOP strategies to identify and molecularly characterize new herpesviruses targeting the DNA polymerase gene. **(A) Conserved sequence domains within herpesvirus DNA polymerases. Functional properties of these domains and amino acid (one letter code) motifs present in the domains are indicated. Motifs chosen as targets for the CODEHOP strategy are shown as black boxes. (B) Schematic diagram of the CODEHOP primer positions, the amplification products and their sizes. See Table 1 for primer sequences.

The *Herpesviridae *was chosen as a target virus family to develop assays to detect and characterize new viral members. All members of the herpesvirus family contain a DNA polymerase within their genome which is highly conserved across the different family members. Multiple alignment of different herpesvirus polymerase sequences revealed blocks of conserved amino acids corresponding to many of the functionally important motifs [16], see Figure 2A. We have developed and refined PCR strategies using CODEHOP PCR primers derived from these conserved sequence blocks to detect novel herpesviruses and characterize their genomes.

**Figure 3:**
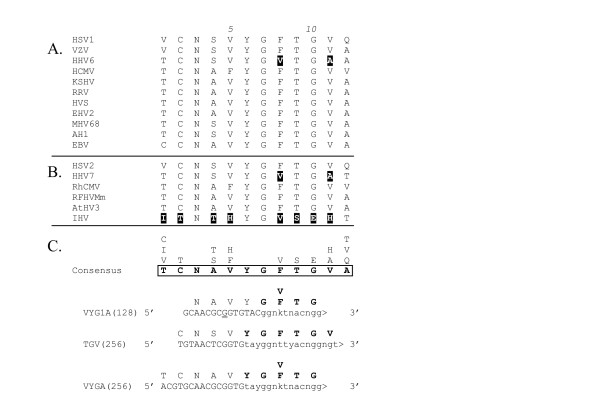
**CODEHOP PCR primers derived from the VYGF/TGV sequence motif. **(A) Multiple sequence alignment of 11 herpesvirus DNA polymerase sequences contained within the conserved VYGF/TGV domain as an output of BlockMaker [32]. (B) Sequences from 6 additional herpesvirus species aligned with the conserved sequence block. (C) The consensus amino acid sequence from the VYGF/TGV motif as determined by the CODEHOP algorithm is presented (in bold and boxed) and the other amino acids found at each position are aligned vertically above the consensus amino acid. The sense-strand "VYG1A" CODEHOP predicted by the CODEHOP software is indicated with the 5' consensus clamp in uppercase and the 3' degenerate core region in lowercase. The sequence, relative position and encoded sequences of the manually designed CODEHOPs, "TGV" and "VYGA" are also shown (see Table 1). Highlighted amino acids are discussed in the text. The degeneracy of the primer pools is indicated in parentheses. DNA polymerase protein sequences were derived from the following herpesvirus species: HSV1, NC_001806; VZV, NC_001348; HHV6, NC_001664; CMV, AF033184; HHV7, NC_001716; RhCMV, AF033184; hCMV, AF033184;; HSV2, NC_001798; RFHVMm, AF005479; MHV68, NC_001826; KSHV, AF005477; HVS, NC_001350; AtHV3, NC_001987; AlHV1, NC_002531; RRV, AF029302; IHV, NC_001493; EBV, NC_001345; EHV2, NC_001650.

**Figure 4:**
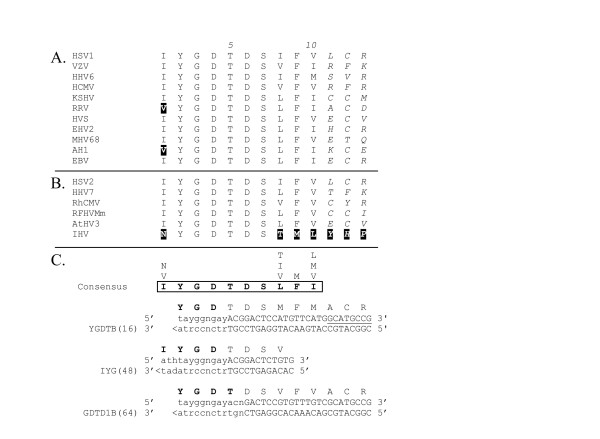
**CODEHOP PCR primers derived from the IYG/GDTD sequence motif **(A)(B) Sequence alignments across the IYG/GDTD motif as described in the legend to Figure 3. (C) The consensus amino acid sequence from the IYG/GDTD motif as determined by the CODEHOP software is presented (in bold and boxed) and the other amino acids found at each position are aligned vertically above the consensus amino acid. The coding strand sequence and the complementary strand corresponding to the "YGDTB" CODEHOP predicted by the CODEHOP algorithm are indicated with the sequences of the 5' consensus clamp in uppercase and the 3' degenerate core region in lowercase. The consensus sequence shows the extent of the sequence block determined by BlockMaker. The CODEHOP algorithm was unable to determine a 5' consensus clamp giving the required Tm due to the small size of the block. Therefore, three additional amino acid positions (in italics) were added to the C' terminal side of the block in (A) and (B) to allow visual inspection of the sequences to manually determine an additional 8 bp of the 5' consensus clamp which are underlined. The nucleotide sequences, relative positions and encoded amino acid sequences for the manually designed CODEHOPs, "IYG" and "GDTD1B" are also shown (see Table 1 for the exact nucleotide sequences of these anti-sense strand primers). The degeneracy of the primer pools is indicated in parentheses and the highlighted residues are discussed in the text. The CODEHOP primers, YGDTB, IYG and GDTD1B are all derived from the antisense DNA strand and are shown below the codons for the sense strand.

**Figure 5:**
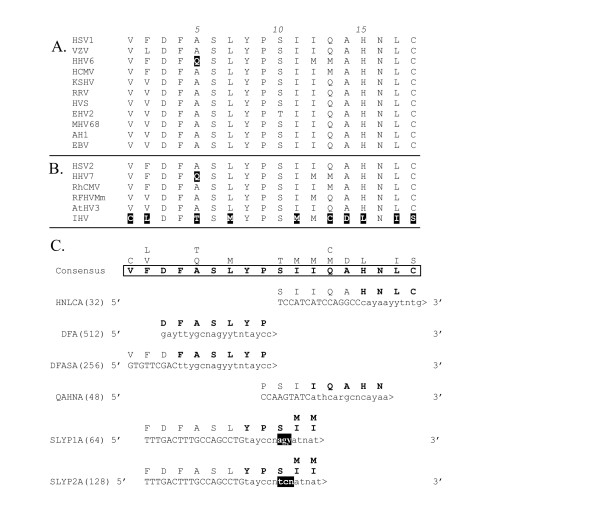
**CODEHOP PCR primers derived from the "DFAS/QAHN" sequence motif **(A)(B) Sequence alignments across the "DFAS" motif as described in the legend to Figure 3. The non-conserved amino acids in the IHV sequence are highlighted (C) The consensus amino acid sequence from the "DFAS" motif as determined by the CODEHOP algorithm is presented (in bold and boxed) and the other amino acids found at each position are aligned vertically above the consensus amino acid. The sense-strand "HNLCA" CODEHOP predicted by the CODEHOP software is indicated with the 5' consensus clamp in uppercase and the 3' degenerate core region in lowercase. The sequence, relative position and encoded sequences of the manually designed CODEHOPs, "DFA", "DFASA", "QAHNA" and "SLYP1A" are also shown (see Table 1). The degeneracy of the primer pools is indicated in parentheses. The codons found in the different herpesvirus sequences encoding the serine (S), block position 6, in the "DFAS" motif were all of the "AGY" type serine codons, so the manually derived primers utilized those codons exclusively at that position.

**Figure 6:**
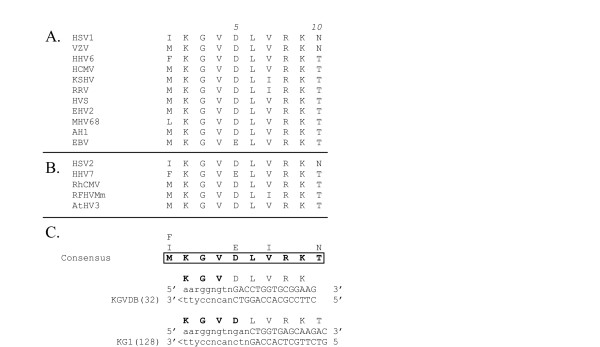
**CODEHOP PCR primers derived from the "KGV" sequence motif **(A)(B) Sequence alignments across the "KGV" motif as described in the legend to Figure 3. (C) The consensus amino acid sequence from the "KGV" motif as determined by the CODEHOP algorithm is presented (in bold and boxed) and the other amino acids found at each position are aligned vertically above the consensus amino acid. The sequences of the coding strand and complementary strand corresponding to the "KGVDB" CODEHOP predicted by the CODEHOP software is indicated. The nucleotide sequences, relative positions and encoded amino acid sequences of the manually designed CODEHOP, "KG1", are also shown (see Table 1 for the exact nucleotide sequences of these anti-sense strand primers). The degeneracy of the primer pools is indicated in parentheses.

Initially, we manually designed a set of nested PCR primers from four of the conserved DNA polymerase blocks (indicated as black boxes in Figure 2A) which could be used to identify new viral polymerases and detect the existence of previously unknown or uncharacterized herpesviruses [8]. The primers, "TGV", "IYG", "DFA" and "KG1" (Table 1), and the blocks of multiply aligned sequences from which the primers were derived are shown in Figures 3, 4, 5, 6, respectively (letters in the primer name refer to conserved amino acids in the sequence motif). Although these primers were alternately referred to as either "consensus" primers or "degenerate" primers within the original publication, all except DFA were designed using the general CODEHOP strategy [2]. In the "TGV-IYG" herpesvirus assay, the "DFA" sense primer was used in an initial PCR amplification with the "KG1" anti-sense primer (Figure 2B). An additional sense primer "ILK" located downstream of the "DFA" motif was also added to the initial amplification reaction [8]. The product from this amplification was used as template in a nested amplification reaction using the "TGV" sense primer and the "IYG" anti-sense primer (Figure 2B). This final PCR product was sequenced to obtain the ~165–180 bp region of the DNA polymerase gene located between the two motifs "TGV" and "IYG". The distance between the two motifs was variable between viral species due to small sequence insertions or deletions.

We have shown the utility of this CODEHOP PCR primer strategy by identifying and characterizing14 previously unknown DNA polymerase sequences from members of the alpha, beta and gamma subfamilies of herpesviruses [8]. Since this original publication, more than 21 additional "TGV-IYG" DNA polymerase sequences from previously uncharacterized herpesviruses have been obtained by other investigators using this CODEHOP primer strategy (see Additional File 1; "TGV-IYG" assay). In some cases, PCR amplification was performed with modified deoxyinosine-substituted primers [17].

**Figure 7:**
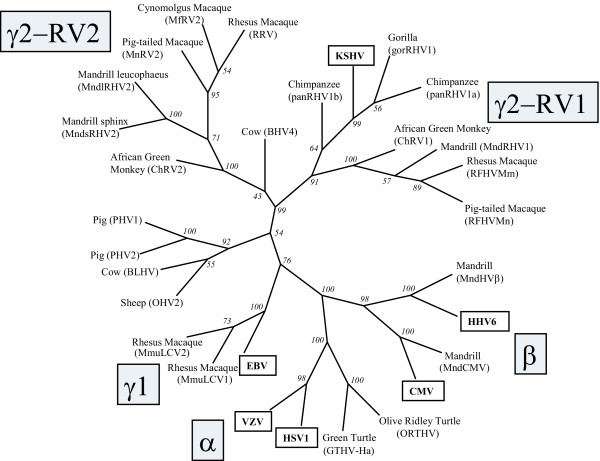
**Phylogenetic analysis of DNA polymerase sequences from different herpesvirus species identified with the "TGV-IYG" CODEHOP assay **The phylogeny of DNA polymerase sequences (~53 amino acids in length) from thirty-six herpesviruses identified using the "TGV-IYG" assay (see Tables 2 and 3) and the corresponding sequences of six representative human herpesviruses (boxed) was determined using the neighbor joining method (Neighbor) applied to pairwise sequence distances (ProtDist) using the Phylip suite of programs [15]. Bootstrap scores (Seqboot) from 100 replicates are indicated and the consensus tree (Consense) is shown. The clustering of the alpha, beta and gamma herpesviruses, including the gamma-1 (*Lymphocryptovirus*) herpesviruses, and the RV1 and RV2 gamma-2 (*Rhadinovirus*) lineages are indicated.

Comparison of the amino acid sequences encoded within the "TGV-IYG" region has allowed phylogenetic comparison of the different herpesvirus species from which these sequences were obtained. Figure 7 shows a phylogenetic tree resulting from the analysis of the sequences obtained from 34 different herpesvirus species identified using the "TGV-IYG" CODEHOP strategy and the corresponding sequences of six representative human herpesviruses. Although the number of amino acid comparisons within this region is limited, ie. only 53 amino acids, preliminary assignment of many of the herpesvirus species to one of the three herpesvirus subfamilies has been possible (Figure 7 and Additional File 1). Values from the bootstrap analysis using 100 replicates are indicated for each branch point. While some of the branch points were not well defined due to the limited amount of sequence data, as indicated by boostrap values less than 50, many groupings were well supported. The analysis shows clearly the grouping of different viral species from evolutionarily related hosts. This is consistent with previous studies which have shown extensive cospeciation of viral species and their host lineages [18].

## Parameters for refinement of the "TVG-IYG" assay

### Limiting degeneracy to increase sensitivity

While the "TVG-IYG" herpesvirus assay demonstrated the ability to detect disparate herpesvirus species in high titer virus cultures *in vitro*, the detection of limiting amounts of virus in tissue samples *in vivo *was problematic. This was especially true in sections obtained from formalin-fixed, paraffin-embedded tissue blocks which contained small amounts of degraded DNA. The degeneracy of the primer pool, ie. the number of different primers necessary to encode all codon possibilities for the specified block of conserved amino acids, plays a direct role in the sensitivity of the PCR amplification. Whereas highly degenerate primers consisting of pools of hundreds or thousands of primers with different DNA sequences may allow amplification of DNA templates present in high copy number, as found in cultured virus stocks, they are less successful in amplifying low copy numbers of DNA templates found in virus infected tissues *in vivo*, especially in formalin-fixed tissue. As the degeneracy increases, the concentration of the primer or primers that will participate in the desired amplification reaction decreases and can become suboptimal. Conversely, the vast excess of primers not participating in the amplification of the targeted gene can cause non-specific amplification which can, in turn, inhibit or mask the amplification of the desired target.

As indicated in Table 1, the degeneracy of the primers utilized in the "TVG-IYG" assay ranged from 48–1024. This level of degeneracy was driven by the number of nucleotide possibilities encoding the targeted amino acids at each position as well as by the number of amino acid positions allowed to be degenerate. Figure 5A shows the DFA/DFAS/QAHN sequence block produced by Block Maker from multiple alignments of 11 different herpesvirus polymerase sequences. Figure 5C shows the consensus amino acids at each position, as determined by the CODEHOP algorithm, which are boxed and bolded with the alternate amino acids positioned above. The original primer manually derived from this motif, "DFA" is, in fact, completely degenerate, with multiple codons provided for each amino acid position, except the ultimate proline (P) residue, yielding a pool of 512 different primers [8]. Because the performance of this primer was consistently suboptimal in samples with limiting template, the overall structure and degeneracy of the primer was altered by designing a PCR primer "DFASA" from the same sequence motif using the CODEHOP methodology. This primer had an 11 bp 5' consensus region and a 3' degenerate core containing multiple codons at 5 amino acid positions resulting in a pool of 256 different primers (Figure 5C). The "DFASA" primer was successfully used to amplify extremely low amounts of viral DNA in a background of genomic DNA from paraffin-embedded formalin-fixed tissue in the discovery of the macaque homolog of Kaposi's sarcoma-associated herpesvirus, called retroperitoneal fibromatosis herpesvirus (RFHV) [9]. Subsequent estimates of virus copy number using real-time quantitative PCR indicated a level of RFHV DNA in the available samples that was 1/100–1/1000 of a single copy cellular gene (unpublished observations). The "DFASA" primer has been successfully used to identify a number of novel alpha-, beta- and gammaherpesviruses in a wide variety of host organisms (see Additional File 1: "DFASA-GDTD1B assay").

Due to the presence of a highly conserved leucine (L) at block position 7 within the "DFAS" motif (Figure 5) which significantly increased the degeneracy of the primer pool with its six possible codons, an additional CODEHOP was designed from the "QAHN" motif immediately downstream of "DFAS" to further decrease degeneracy. The "QAHNA" primer had an 11 bp 5'consensus region and a 3' degenerate core containing multiple codons at 4 amino acid positions resulting in a pool of 48 different primers (Figure 5C). This CODEHOP has been successfully used to identify several primate rhadinoviruses related to KSHV in tissue samples with limiting amount of viral DNA [10,19], see also Additional File 1.

### Primer bias and specificity

**Figure 8:**
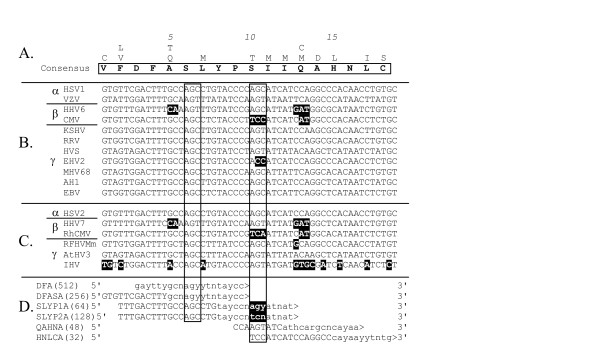
**Alignment of CODEHOP PCR primers with the nucleotide sequences encoding the "DFAS/QAHN" sequence block **(A) Amino acid consensus sequence – see Figure 5C (B) Nucleotide sequences encoding the amino acids in the "DFAS/QAHN" sequence block from the 11 different herpesvirus species that were used to generate the sequence block. (C) Nucleotide sequences from six additional herpesvirus species. (D) Nucleotide sequences of five manually designed primers "DFA", "DFASA", "SLYP1A", "SLYP2A and "QAHNA", and a primer designed using the CODEHOP software (HNLCA). The codons from two conserved serine positions are boxed and nucleotide sequences mismatched with the different 3' degenerate cores of the primers are highlighted in black. The subfamily associations of the different viral species are indicated.

The primers developed for the "TGV-IYG" assay were designed to amplify polymerase fragments from herpesviruses of all three subfamilies based on conserved motifs within the known sequences. However, very few sequence motifs were absolutely conserved between the most divergent herpesviruses. For example, the catfish ictalurid herpesvirus (IHV) lacked the "KGV" motif from which the initial "KGV" primer was derived (Figure 6). Furthermore, numerous sequence differences were present in the IHV DNA polymerase within the DFAS/QAHN motif which was otherwise highly conserved in other herpesvirus species (highlighted residues in Fig. 5B). Because of these differences, the IHV sequence was excluded from the primer design of the "DFA", "DFASA" and "QAHNA" PCR primers. As shown in Figure 5C, the "DFA" and "DFASA" primers have mismatches with the IHV sequence at the alanine (A) and leucine (L) codons (Block positions 5 and 7, respectively; Figure 5B) and the "QAHNA" primer mismatches at three codon positions (Block positions 13–15; Figure 5B), all within the 3' degenerate cores. Figure 8 shows the presence of nucleotide mismatches with the IHV sequence throughout the different primers (black highlighting). Thus, the lack of the "KGV" motif and sequence differences in the "DFA" primer strongly biased the "TGV-IYG" assay against IHV-like herpesvirus sequences. In order to identify IHV-like herpesviruses, new primers would have to incorporate these sequence differences.

The "DFA" and "DFASA" primer pools were originally designed using only the alanine (A) codon at block position 5 in the "DFAS" motif and did not include the glutamine (Q) codon found in that position of the motif in HHV6 and HHV7, "DFQS" (highlighted, Figure 5A, B). The nucleotide mismatches in this region are shown in Figure 8. While the "DFA" and "DFASA" primers are biased by design against HHV6 and HHV7, they have been used successfully to detect betaherpesviruses related to HHV6 and HHV7 [8]. This suggests that mismatches 13–14 nucleotides from the 3' end of the primer, do not have major affects on the utility of the primers, especially when viral template is not limiting.

More significant bias against HHV6- and HHV7-like herpesviruses was present in the "TGV" primer used in conjunction with the "IYG" primer in the secondary nested PCR reaction in the "TGV-IYG" assay (see Figure 2B). The "TGV" primer contains the partial valine (V) codon "GT" at its 3' end (Block position 11; Figure 3C). Since both HHV6 and HHV7 contain alanine (A) (codon = GCN) at this position (highlighted in Fig. 3A, B), the "TGV" primer would mismatch at the 3' terminal nucleotide with both HHV6- and HHV7-like sequences. This mismatch occurs at the 3' end of the "TGV" primer and is predicted to significantly impair polymerase extension. To remove this bias, the "TGV" primer was redesigned as the "VYGA" primer removing the 3' terminal "GT" of the valine codon and the terminal degenerate position of the glycine (G) codon. The "TGV" primer contained an additional bias against amplification of HHV6-like sequences due to the use of only the phenylalanine (F) codons (TTY) (Block position 8) at a position encoding valine (V) in both HHV6 and HHV7 (highlighted in Figure 3A and 3B). To remove this bias, "VYGA" was designed to include both the valine (V) and (F) codons at this position. The total degeneracy of the "TGV" and "VYGA" primer pools remained the same, with 256 different primers, due to the loss of the degenerate codon position in the glycine, block position 10 in "TGV" and the gain of the degenerate codon positions in the valine, block position 8 in "VYGA".

The subsequent cloning and sequence analysis of new herpesvirus DNA polymerases from the rhadinoviruses, rhesus rhadinovirus (RRV) and alcelaphine herpesvirus 1 (AlHV1) [20,21], revealed mismatches with the downstream "IYG" primer of the "TVG-IYG" herpesvirus assay. The "IYG" primer (a reverse orientation primer) includes the codons (ATH) for isoleucine (I) at its 3' end (Block position 1; Figure 4C). Both RRV and AH1 contain a valine (V) codon (GTN) at this position (highlighted in Figure 4A). Thus, "IYG" is biased against RRV-like or AH1-like rhadinoviruses due to a T-C mismatch at the 3' end of the primer. To eliminate this bias, the "IYG" primer was redesigned as "GDTD1B" to remove the isoleucine position within the 3' degenerate core (Figure 4C) and, in addition, the length of the 5' consensus clamp was increased.

### Decrease in size of the amplification products

Because typical tissue samples especially paraffin-embedded formalin-fixed tissue contain degraded DNA with sizes averaging near 300–500 bp in length, we decided to decrease the maximal amplification product size of the herpesvirus assay. The initial amplification product of the "TGV-IYG" assay (DFA-KG1) was ~800 bp (Fig. 2B). To reduce the initial amplification product size, a hemi-nested PCR assay was developed in which the newly designed downstream anti-sense primer "GDTD1B" targeting the highly conserved "YGDT" motif was used in a primary PCR amplification with the new upstream primer "DFASA". This amplification yields an approximate 500 bp PCR product (Figure 2B). This initial PCR product is then used as template in a secondary PCR amplification using the nested primer "VYGA" with the downstream anti-sense primer "GDTD1B". This amplification yields a PCR product of approximately 200 bp (see Figure 2B). These modifications produce amplification products close to the average size of degraded DNA present in fixed tissue.

## The "DFASA/QAHNA-GDTD1B" herpesvirus assay: a refinement of the "TGV-IYG" assay

**Figure 9:**
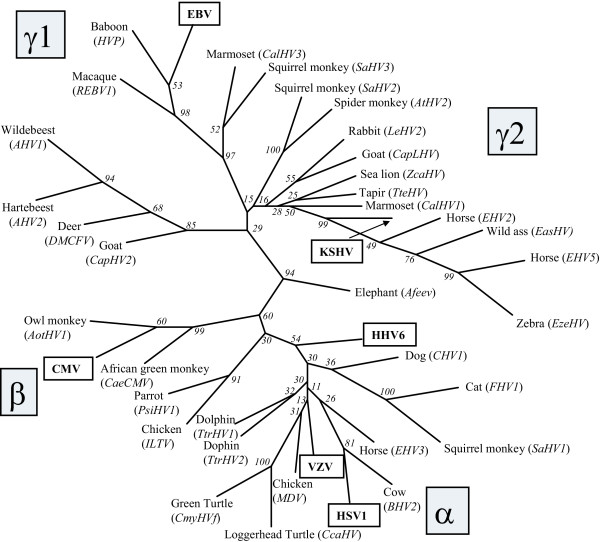
**Phylogenetic analysis of DNA polymerase sequences from different herpesvirus species identified with CODEHOP assays targeting the DFAS and YGDT motifs **The phylogeny of DNA polymerase sequences (~142 amino acids in length) from 25 different herpesvirus species identified using either the "DFA-IYG", "DFASA-GDTD1B", or QAHNA-GDTD1B assays (see Tables 2 and 3), was determined as described in the legend to Figure 7.

We have developed a refined herpesvirus assay using the optimized DNA polymerase CODEHOP PCR primers, discussed above. This assay was designed to use only three CODEHOPs in a hemi-nested PCR assay in which "DFASA" and "GDTD1B" are used in an initial PCR amplification (Figure 2B). The product from that amplification is used as template in a secondary amplification with "VYGA" and the original anti-sense primer "GDTD1B". A variation of this assay uses the "QAHNA" to replace "DFASA". Thus, the amplification of novel polymerase sequences required the conservation of only three motifs, rather than five in the original "TGV-IYG" assay. Using these assays, we have identified three novel homologs of the newly characterized human herpesvirus, KSHV, in two species of macaques [9] (see Table 1, RFHVMn, RFHVMm and MneRV2). Phylogenetic analysis of the molecular sequences obtained from these studies provided strong evidence for the existence of two distinct lineages of γ2 rhadinoviruses related to KSHV, called rhadinovirus-1 (RV1) and rhadinovirus-2 (RV2) (Figure 9) [10]. Subsequent studies by others using this assay, have identified the presence of additional members of these two lineages in other Old World primates, including African green monkeys [19], mandrills [22], chimpanzees [23,24] and gorillas [24] (see Additional File 1). This data predicts the existence of another human herpesvirus closely related to KSHV belonging to the RV-2 lineage of rhadinoviruses [10].

The utility of the "DFASA/QAHNA-GDTD1B" assays has been demonstrated by these and other studies in which more than 19 novel herpesviruses from the alpha, beta and gamma subfamilies of a wide variety of host species have been identified and molecularly characterized using CODEHOPs (Tables 2 and 3). Comparison of the amino acid sequences encoded between the "DFAS" and "IYG/GDTD" motifs has allowed the phylogenetic comparison of the different herpesvirus species from which these sequences were obtained. Figure 9 shows a phylogenetic tree resulting from the analysis of the sequences obtained from the "DFA-IYG", and "DFASA/QAHNA-GDTD1B" assays and the corresponding sequences of six representative human herpesviruses. Multiple sequence alignments of the viral sequences were performed and the positions containing gaps were eliminated, leaving 142 amino acid positions for comparison. These sequences were analyzed using protein distances and neighbor-joining analysis implemented in the Phylip analysis package [15]. As shown in Figure 9, most of the different viral species could be unambiguously included within either of the three herpesvirus subfamilies as indicated by the high bootstrap scores obtained for most of the branch points. However, the positioning of the branch points for certain viral species could not be reliably determined using the available sequence information. Such uncertainty has been seen in similar analysis of specific herpesvirus species using much larger data sets [18]. The results obtained using the 142 amino acid comparisons confirmed and extended the phylogenic relationships predicted from the "TVG-IYG" results derived from only 53 amino acid comparisons. Furthermore, the phylogenetic relationships predicted by the different CODEHOP assays have been subsequently confirmed when substantially more sequence information was obtained from the new viral species, see [10,11]. The phylogenetic relationships shown in Figure 9 are consistent with the findings that extensive cospeciation of viral species and their host lineages has occurred during evolution [18]. The wide variety of different herpesvirus species identified using the CODEHOPs assays targeting the DNA polymerase gene, as shown in Figures 7 and 9, indicate the wide applicability of the CODEHOPs assays to detect herpesviruses from disparate host lineages.

**Table 2:** Alpha- and Betaherpesviruses identified and/or characterized using CODEHOP-based PCR assays targeting the herpesvirus DNA polymerase

**Virus species**^1^	**Abbrev.**^1^	**Host**	**Strain**	**Assay**	**Accession (#aa)**	**Reference**
**Alphaherpesvirus**						
Bovine HV-2	BHV2	Cow		TGV-IYG^2^	AAC59453 (59aa)	[36]
Canid HV-1	CHV1	Dog	D004	TGV-IYG	AAC55646 (60aa)	[8]
Caretta caretta HV	CcaHV	Florida loggerhead turtle		TGV-IYG	AAD24564 (60aa)	[37]
Chelonia mydas HV-Florida	CmyHVf	Florida green turtle		TGV-IYG DFASA-GDTD1B^3^	AAD24565 (60aa) AAC26682 (161aa)	[37] [38]
Chelonia mydas HV-Hawaii	CmyHVh	Hawaiin green turtle		DFASA-GDTD1B	AAC26681 (161aa)	[38]
Equid HV-3	EHV3	Horse	C-175	TGV-IYG	AAD30140 (59aa)	[17]
Felid HV-1	FHV1	Cat	C-27	TGV-IYG	AAC55649 (60aa)	[8]
Infectious laryngotracheitis virus (Gallid HV-1)	ILTV	Chicken	N-71851	TGV-IYG	AAC55650 (59aa)	[8]
Marek's disease virus (Gallid HV-3)	MDV	Chicken	GA5	TGV-IYG	AAC55651 (59aa)	[8]
Lepidochelys olivacea HV	LolHV	Olive ridley turtle		DFASA-GDTD1B	AAC26684 (161aa)	[38]
Psittacid HV-1	PsiHV1	Parrot	RSL-1	TGV-IYG	AAC55656 (59aa)	[8]
Saimiriine HV-1	SaHV1	S. American squirrel monkey	MV-5-4	TGV-IYG	AAC55657 (60aa)	[8]
Tursiops truncatus HV-1	TtrHV1	Bottlenose dolphin	Heart	TGV-IYG^2^	AAF62170 (60aa)	Unpublished
Tursiops truncatus HV-2	TtrHV2	Bottlenose dolphin	Lung	TGV-IYG	AAF07208 (63aa)	Unpublished
**Betaherpesvirus**						
African elephant endotheliolytic virus	Afeev	African elephant	Case 2	TGV-IYG	AAD24549 (60aa)	[39]
Asian elephant endotheliolytic virus	Aseev	Asian elephant	Case 3	TGV-IYG	Not Deposited (60aa)	[39]
Aotine HV-1	AoHV1	Owl monkey	S43E	TGV-IYG	AAC55643 (57aa)	[8]
Chlorocebus aethiops cytomegalovirus (Cercopithecine HV-5)	CaeCMV	African green monkey	CSG	TGV-IYG	AAC55647 (57aa)	[8]
Mandrill cytomegalovirus	MndCMV	Mandrill leucophaeus	Mnd205	DFASA-GDTD1B	AAG39064 (157aa)	[22]
Mandrill HV β	MndHVβ	Mandrill sphinx	Mnd301	DFASA-GDTD1B	AAG39065 (159aa)	[22]

^1^Names and abbreviations are usually derived from the original publications although some have been modified to conform to a three letter code derived from the first letter of the genus and the first two letters of the species, ie. *Macaca mulatta *= *Mmu*. This was necessary due to the number of different viral species and hosts which could not be distinguished with a two letter code.

^2^Reference [8]

^3 ^Reference [9]

^4 ^Reference [17]

^5 ^Reference [10]

^6 ^Primers modified (see reference)

**Table 3:** Gammaherpesviruses identified and/or characterized using CODEHOP-based PCR assays targeting the herpesvirus DNA polymerase (see legend to Table 2)

**Virus species**^1^	**Abbrev.**^1^	**Host**	**Strain**	**Assay**	**Accession (#aa)**	**Reference**
**Gammaherpesvirus-1**						
Bovine lymphotrophic HV	BLHV	Cow		DFA-IYG^4^	AAC59451 (160aa)	[36]
Callitrichine HV-3	CalHV3	Marmoset		TGV-IYG	AAF05882 (58aa)	Unpublished
Leoporid HV-2	LeHV2	Rabbit		TGV-IYG	AAC55655 (54aa)	[8]
Rhesus lymphocryptovirus-1 (cercopithecine HV-15)	MmuLCV1	Macaque mulatta		DFASA-GDTD1B TGV-IYG	This study AF091053	This study Unpublished
Rhesus lymphocryptovirus-2	MmuLCV2	Macaque mulatta		DFASA-GDTD1B		This study
HV papio (cercopithecine HV-12)	HVP	Baboon		TGV-IYG	AAF05878 (58aa)	Unpublished
Ovine HV 2	OHV2	Sheep		DFA-IYG	AAC59455 (161aa)	[36]
Porcine lymphotrophic virus-1a	PLHV1a	Pig	56	DFA-IYG	AAD26258 (155aa)	[40]
Porcine lymphotrophic virus-1b	PLHV1b	Pig	68	DFA-IYG	AAD26257 (155aa)	[40]
Saimiriine HV-3	SaHV3	S. American squirrel monkey		TGV-IYG	AAF98285 (57aa)	Unpublished
Zalophus californianus HV	ZcaHV	Sea lion		TGV-IYG	AAF07188 (55aa)	Unpublished
**Gammaherpesvirus-2**						
Alcelaphine HV-1	AlHV1	Wildebeest		TGV-IYG	AAC59452 (58aa)	[36]
Alcelaphine HV-2	AlHV2	Hartebeest		TGV-IYG	AAG21352 (58aa)	Unpublished
Caprine HV-2	CapHV2	Goat		TGV-IYG	AAG21351 (59aa)	Unpublished
Caprine lymphotropic HV	CapLHV	Goat		TGV-IYG	AAG10783 (58aa)	Unpublished
Deer malignant catarrhal fever virus	DMCFV	Deer		TGV-IYG	AAD56945 (59aa)	[41]
Ateline HV-2	AtHV2	S. American spider monkey		TGV-IYG	AAC55644 (55aa)	[8]
Bovine HV-4	BHV4	Cow		DFA-IYG	AAC59454 (156aa)	[36]
Callitrichine HV-1	CalHV1	Marmoset		TGV-IYG	AAC55645 (55aa)	[8]
Chlorocebus rhadinovirus-1	ChRV1	African green monkey	Z8	QAHNA-GDTD1B^5^	CAB61753 (151aa)	[19]
Chlorocebus rhadinovirus-2	ChRV2	African green monkey	L1	QAHNA-GDTD1B	CAB61754 (151aa)	[19]
Equine HV-2	EHV2	Horse		TGV-IYG	AAC55648 (55aa)	[8]
Equine HV-5	EHV5	Horse		TGV-IYG^6^	AAD30141 (56aa)	[17]
Gorilla rhadinoherpesvirus 1	gorRHV1	Gorilla	GorGabOmo	DFASA-GDTD1B	AAG23218 (158aa)	[24]
Kaposi's sarcoma-associated HV (HHV8)	KSHV	Human	KS187	DFASA-GDTD1B	AAC57974 (151aa)	[9] [10]
Macaque fascicularis rhadinovirus-2 (Macaque fascicularis gamma virus)	MfaRV2	Macaque fascicularis		DFASA-GDTD1B	AAF23082 (158aa)	[42]
Macaque nemestrina rhadinovirus-2	MneRV2	Macaque nemestrina	Mne442N	DFASA-GDTD1B	AAF81664 (158aa)	[10]
Mandrill rhadinoherpesvirus-1	MndRHV1	Mandrill sphinx	Mnd15	DFASA-GDTD1B	AAG39066 (158aa)	[22]
Mandrill rhadinoherpesvirus-2	MndlRHV2	Mandrill leucophaeus	Mnd205	DFASA-GDTD1B	AAG39061 (158aa)	[22]
Mandrill rhadinoherpesvirus-2	MndsRHV2	Mandrill sphinx	Mnd13	DFASA-GDTD1B	AAG39060 (158aa)	[22]
Pan troglodytes rhadinoherpesvirus-1a	panRHV1a	Chimpanzee	PanCamDja	DFASA-GDTD1B	AAG23140 (158aa)	[24]
Pan troglodytes rhadinoherpesvirus-1b	panRHV1b	Chimpanzee	PanCamEko	DFASA-GDTD1B	AAG23142 (158aa)	[24]
Retroperitoneal fibromatosis HVMm	RFHVMm	Macaque mulatta	MmuYN91-224	QAHNA-GDTD1B	AAC57976 (151aa)	[9] [10]
Retroperitoneal fibromatosis HVMn	RFHVMn	Macaque nemestrina	Mne442N	DFASA-GDTD1B	AAF81662 (158aa)	[9] [10]
Rhesus rhadinovirus (Macaque mulatta gamma virus)	RRV	Macaque mulatta		DFASA-GDTD1B	AAF23083 (158aa)	[42]
Tapirus terrestris HV	TteHV	Tapir		TGV-IYG^6^	AAD30142 (55aa)	[17]
Equus somalicus HV	EsoHV	Wild ass		TGV-IYG^6^	AAD30143 (57aa)	[17]
Equus zebra HV	EzeHV	Zebra		TGV-IYG^6^	AAD30144 (55aa)	[17]

## The "SLYP1A-GDTD1B" herpesvirus assay: a general herpesvirus detection assay

We designed additional primers from the DFAS/QAHN sequence motif using the CODEHOP strategy to develop further assays to detect new herpesviruses. The primer "SLYP1A" was one such primer designed to eliminate bias in the 3' degenerate core of "DFA" and "DFASA" primers against HHV6 and HHV7, described above. The "SLYP1A" primer overlaps the "DFA" and "DFASA" primers and extends further downstream in a region very well conserved across the different herpesvirus species including HHV6 and HHV7 (Block positions 8–12; Figure 5C) [10]. Primer design across this region was based on the similarities in the first two positions for the codons for isoleucine (I) – (ATA, ATC, ATT) and methionine (M) – (ATG). These two amino acids are conserved in two positions within this sequence block in all herpesvirus species, including IHV (Block positions 11,12; Figure 5) and provide the penultimate and ultimate 3' codons for the primer. Also, the SLYP1A primer was designed with only one of the two codon types utilized for serine (S) – (AGY) to minimize degeneracy in the 3' degenerate core (Block position 10; Figure 5C). Serine at this position (Block position 10; Figure 8) is encoded by AGY-type codons in all herpesvirus species, except for CMV-like herpesviruses which use TCN-type codons and EHV2 which contains a codon for threonine. A second related primer, SLYP2A was also designed from this region with an identical sequence except that the other serine codons (TCN) were used in the third position. Although this primer was biased for CMV-like sequences, we have successfully amplified KSHV which contains an AGT codon (unpublished results).

**Figure 10:**
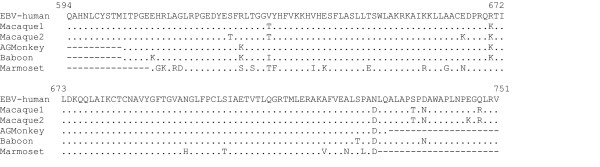
**Amino acid sequence comparision of two rhesus macaque EBV homologs detected using the "SLYP1A-GDTD1B" CODEHOP assay **Positions with identity to human EBV are shown as a (.), and unidentified flanking regions or inserted gaps are indicated as (-). Numbering is from the human EBV DNA polymerase sequence. *M. mulatta*-1 and *M. mulatta*-2 sequences are listed in Table 1 as MmuLCV1 and MmuLCV2. The *Macaca fascicularis*, African green monkey (*Chlorocebus aethiops*) and baboon (*Papio hamadryas*) EBV-like sequences were published in [33] but not deposited in Genbank. The marmoset EBV-like sequence was deposited in Genbank as a AF291653 [34].

We have previously used "SLYP1A" and "GDTD1B" to identify a new herpesvirus related to RRV, called *Macaca nemestrina *rhadinovirus-2 (MneRV2) in spleen tissue [10]. We subsequently used this assay to screen for herpesviruses in lymphomas from two rhesus macaques, L758 and 881, from the Tulane Regional Primate Research Center. DNA was kindly provided by LS Levy. Strong PCR products were obtained in primary amplification reactions and were cloned and sequenced. The lymphoma from rhesus 881 yielded clones containing a single sequence which was highly related to human EBV. From the lymphoma from rhesus L758, we obtained two distinct EBV-like sequences, one which was identical to the first lymphoma sequence and the other one which contained 10 nucleotide differences across the 475 bp fragment (98% identity). Analysis of the encoded amino acids revealed 3 amino acid differences (98% identity) between the two rhesus EBV-like sequences (MmuLCV1 and MmuLCV2) (Figure 10). These sequences clustered closely with human EBV in the γ1 branch of the phylogenetic tree shown in Figure 9. The identification of DNA polymerases from two types of EBV-like lymphocryptoviruses corroborates previous reports of the existence of two closely related lymphocryptoviruses in rhesus macaques [25] identified by sequence comparision of two distinct EBNA-2 genes. This is similar to the situation in humans where two different EBV species, EBV1 and EBV2 have been identified [26].

## Using the CODEHOP strategy to determine the complete sequence of novel viral genes

**Figure 11:**
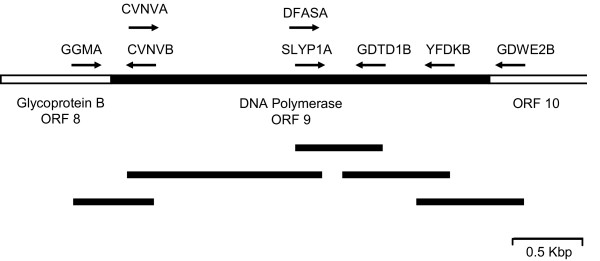
**CODEHOP strategy to determine the complete sequence of a gammaherpesvirus DNA polymerase gene **The conserved linear order of the DNA polymerase gene, ie ORF 9, and the ORF 8 and ORF 10 flanking genes, characteristic of gammaherpesviruses, is shown. The position of the CODEHOP PCR primers used to obtain the sequence of the entire DNA polymerase gene of RFHVMn and RFHVMm is shown. The overlapping PCR products obtained using the CODEHOP and gene-specific primers are shown.

The CODEHOP assays described above targeted a restricted region of one gene and only provided limited sequence information. We have also used CODEHOPs to obtain the complete sequence of targeted genes and identify flanking genes within the unknown viral genome. To obtain the complete sequences of the DNA polymerase genes of the newly identified herpesvirus species of macaques, RFHVMn and RFHVMm, we designed CODEHOP PCR primers from additional conserved sequence blocks within the DNA polymerase (Figure 11 and Table 4). The new DNA polymerase-derived CODEHOP PCR primers, "CVNVA" and "YFDKB" were used in conjunction with gene specific primers derived from within the sequence of the original CODEHOP PCR product "DFASA-GDTD1B to obtain overlapping PCR products across the majority of the DNA polymerase gene [10]. In all gammaherpesviruses, the DNA polymerase gene (ORF 9) is flanked upstream by ORF 8, the glycoprotein B, the most highly conserved glycoprotein in herpesviruses and downstream by ORF 10, a gene conserved within the gammaherpesviruses with unknown function (Figure 11). CODEHOPs were designed from conserved sequence blocks present in ORF 8 – "FREYA" and "GGMA" and in ORF 10 "GDWE2B" (Table 4). Using a combination of gene-specific primers obtained from the DNA polymerase sequence obtained above and the new CODEHOPs derived from flanking regions, overlapping PCR products spanning 331 bp of the glycoprotein B genes, 3,039 bp of the DNA polymerase genes, and 27 bp of the ORF 10 gene homolog were obtained for RFHVMn and RFHVMm [10].

**Table 4:** CODEHOP and gene-specific primers developed for cloning the complete DNA polymerase gene of novel macaque rhadinoviruses.

Primer	Gene Target	Bias		Sense	5'>3' Sequence (degenerate codons are in lower case)^1^
				
		3' Core	5' Clamp		
**CODEHOP**^2^					
FREYA (32)	gB^4^	γHV^3^	KSHV^4^	+	TTTGACCTGGAGACTATGttymgngartayaa
GGMA (128)	gB	γHV	KSHV	+	ACCTTCATCAAAAATCCCttnggnggnatgyt
CVNVA (64)	DNA pol	γHV	KSHV	+	GACGACCGCAGCGTGTGCGTGaaygtnttyggnca
CVNVB (64)	DNA pol	γHV	KSHV	-	TAAAAGTACAGCTCCTGCCCGaanacrttnacrca
YFDKB (16)	DNA pol	γHV	KSHV	-	TTAGCTACTCCGTGGAGCagyttrtcraarta
GDWE2B (8)	ORF 10	γHV	KSHV	-	GAAGTGGCAGTTGGAGAGGCTGACCTCCcartcncc

^1 ^See legend to Table 1 for the I.U.B. code.

^2 ^See Figure 11 for the relative positions of the conserved sequence blocks from which the CODEHOPs were derived. The degree of degeneracy, ie the number of individual primers in the pool, is given in parentheses.

^3 ^The CODEHOPs were derived from the alignment of conserved genes within the gammaherpesvirus subfamily.

^4 ^The 5' Clamp region was derived from the KSHV sequence flanking the 3' core in order to target genes from RFHV, the macaque homolog of KSHV.

## Using the CODEHOP strategy to characterize genomic regions within novel viral genomes

**Figure 12:**
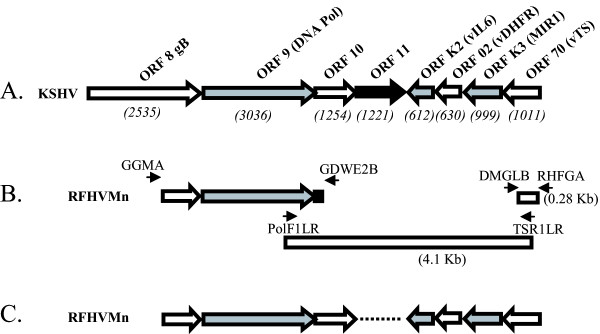
**CODEHOP strategy to determine the complete sequence of a region of the divergent locus B of a macaque homolog of KSHV**. A) the linear order of genes within the divergent locus B of KSHV [35]. Gene size in bp is shown in parantheses. B) The positions of the CODEHOP PCR primers used to obtain the DNA polymerase (GGMA/GDWE2B: see Figure 11) and thymidylate synthase (TS) (DMGLB/RHFGA) sequences are shown. The gene specific primers from the DNA polymerase (PolF1LR) and TS (TSR1LR) genes used in long range PCR are indicated. C) the linear order of genes within the divergent locus B of RFHVMn determined by the CODEHOP technique [11].

Often the linear order of genes within the genomes of related viruses is maintained. Thus, the spacing and orientation of specific genes can be predicted in the genomes of related novel viruses. CODEHOP PCR primers can be utilized to obtain sequences within conserved genes which flank a targeted genomic region. Gene-specific PCR primers derived from these sequences can then used in long-range PCR to obtain the sequence of the entire genomic region between the flanking genes. We have utilized this approach to clone and characterize a portion of the divergent locus B of the genome of the macaque rhadinovirus, RFHVMn [11]. Divergent locus B was identified in KSHV and other rhadinoviruses and contains a number of viral homologs of cellular genes that have been captured during virus evolution [27]. Part of the divergent locus B of KSHV extends upstream of the ORF 9 DNA polymerase gene to a viral homolog of the thymidylate synthase (TS) gene situated approximately 4 kb away (Figure 12A). TS is a cellular gene and a non-functional pseudogene is present in humans. Viral TS homologs are well conserved and are found in several herpesvirus species, including KSHV, VZV, EHV2, HVS and AtHV3. To characterize the putative divergent locus B between the DNA polymerase and TS genes of RFHVMn, we targeted the TS gene for PCR amplification using the CODEHOP approach.

**Figure 13:**
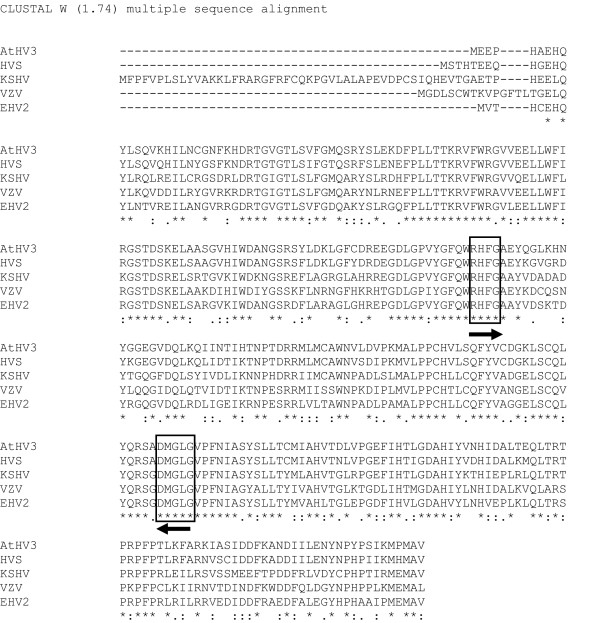
**ClustalW alignment of multiple herpesvirus TS sequences**. The ClustalW output was obtained from the five TS sequences shown in Figure 15. The conserved "RHFG" and "DMGL" motifs which were chosen as targets in the design of the RHFGA (sense orientation) and DMGLB, DMGLXB and DMGLX1B (anti-sense orientation) CODEHOP PCR primers are indicated.

Two conserved blocks of amino acids within the TS gene family containing 10 and 11 identical amino acids were chosen as candidates for CODEHOP design. The 10 amino acid "RHFG" upstream motif (Fig. 13) is completely conserved between the viral sequences, the human sequence and the human TS pseudogene. The 11 amino acid "DMGL" downstream motif (Fig. 13) while completely conserved between the viral and human sequences is not present in the cellular TS pseudogene (data not shown). Since the two motifs in the cellular TS gene are separated from each other by a large intron, CODEHOP PCR amplification of DNA containing a mixture of viral and cellular DNA should only produce a virus-specific ~280 bp PCR product (Fig. 12B).

**Figure 14:**
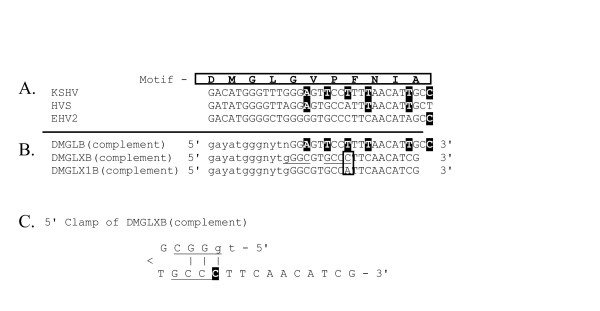
**Alignment of CODEHOPs with the nucleotide sequences of the "DMGL" motif in several herpesvirus TS genes**. A) Nucleotide sequences encoding the "DMGL" motif in several rhadinoviruses. B) Complementary sequences of CODEHOP PCR primers derived from the "DMGL" motif. The sequence of the complementary strand of the primer is shown to identify the coding sequence. The actual PCR primer is the complement of the sequence. DMGLB was biased towards KSHV-like sequences by using the codons from the KSHV TS gene in the 5' clamp region of the primer with KSHV-specific nucleotides highlighted (3' region of the complementary coding strand shown). DMGLXB was predicted from the amino acid sequence block of the conserved "DMGL" motif using the CODEHOP software and utilizes the most common human codons for the amino acids in the 5' clamp region, and is unbiased in design. The underlined sequence in the 5' clamp region can form a stem-loop structure, shown in C. The CODEHOP PCR primer, DMGLX1B, is a revised version of DMGLXB to eliminate base pairing in the stem-loop structure by changing the highlighted cytosine (C) in Fig. 13-C. to an adenosine (A), boxed in Fig. 13-B.

The design of the "DMGLB" CODEHOP from the conserved "DMGL" motif is shown in Figure 14. This primer was designed before the CODEHOP prediction program was available. Because RFHVMn is closely related to the gammaherpesvirus, KSHV, the "DMGLB" CODEHOP was biased towards gammaherpesviruses, in particular KSHV-like herpesviruses, in order to target the RFHV genomes. In Figure 14, the nucleotide sequences encoding the "DMGL" motif from the TS genes of KSHV, HVS and EHV2 were multiply aligned with the encoded amino acid sequence. Because "DMGL" was the downstream motif, the "DMGLB" CODEHOP was designed to be antisense, however, the complementary sequence of the primer is shown to identify codons (Figure 14). Thus, the degenerate core of the CODEHOP spans the codons for the aspartic acid (D), methionine (M), glycine (G), and leucine (L) of the motif, and is indicated in lower case letters in Figure 14B. The degenerate core provides all possibilities of the codons for these four conserved amino acids and thus has no bias. However, the nucleotides within the consensus region, shown in capitol letters, were chosen at each codon position to be similar to the sequence of KSHV (highlighted in Figure 14A), thus biasing the primer towards KSHV-like sequences.

The TS targeted CODEHOPs "DMGLB" and "RHFGA" (see Table 5) were used in PCR amplification reactions with DNA isolated from retroperitoneal fibromatosis (RF) tumor tissue of a pig-tailed macaque, *Macaca nemestrina*, as described previously [10]. A PCR product of the predicted size (280 bp) was obtained and cloned and sequenced, see Fig. 12B. The sequence was 68% identical to the KSHV TS sequence and 64% identical to the TS sequence of RRV, a more distantly related gammaherpesvirus. A TS-specific primer, TSR1LR, derived from this sequence and a DNA polymerase-specific primer, PolF1LR, were chosen to amplify the region between the DNA polymerase and TS genes of RFHV (Table 5 and Figure 12B). Long range PCR amplification produced a PCR product of ~4.1 kb which was sequenced. The linear order and sequence of 5 novel genes present in the diverse region B of the RFHVMn virus was obtained (Figure 12C). Although region B of RFHV lacked a homolog of KSHV ORF 11, homologs of all the other KSHV genes in this region were present and in the same order within the genome [10].

**Table 5:** CODEHOP and gene-specific primers developed for cloning the divergent region B within the RFHV genome

Primer	Gene Target	Bias^2^		Sense	5'>3' Sequence (degenerate codons are in lower case)^3^
				
		3' Core	5' Clamp		
**CODEHOP**^1^					
RHFGA (48)	TS^4^gene	All cellular and viral TS	KSHV^5^	+	CCTGTTTACGGTTTCcartggagrcayttygg
DMGLB (32)	TS gene	All cellular and viral TS	KSHV	-	GGCAATGTTAAAAGGAACTccnarncccatrtc
**RFHVMn-specific**^6^					
PolF1LR	DNA polymerase	NA^7^	NA	+	CCACCGTCCCAGACCAACGAAAGCGCCAGA
TSR1LR	TS gene	NA	NA	+	GTCTGCCTGGAATCCCGTGGATATACCAAA

^1 ^CODEHOP, consensus-degenerate hybrid oligonucleotide primers. The degree of degeneracy, ie the number of individual primers in the pool, is given in parentheses.

^2 ^Bias indicates the reliance on a specified subset of sequences for determination of the 3' degenerate core or 5' consensus clamp.

^3 ^See legend to Table 1 for the IUB code.

^4 ^TS, thymidylate synthase.

^5 ^Clamp region derived from the KSHV viral TS gene [11]

^6 ^Primer sequence derived from the RFHVMn sequence obtained by the CODEHOP technique

^7 ^NA, not applicable – these are gene-specific primer

## CODEHOP-mediated PCR – a general approach to identify novel viral genes

**Figure 15:**
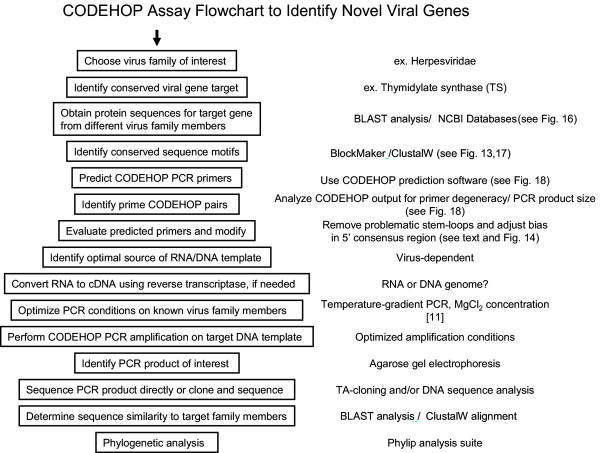
**CODEHOP assay flowchart to identify novel viral genes**. The general approach to use CODEHOP-mediated PCR to identify novel viral genomes from a target virus family is shown schematically with links to specific software sites.

In the previous sections of this review the CODEHOP assays and PCR primers that we have used to identify and characterize novel herpesvirus genes and genomes have been discussed in detail. However, CODEHOP-mediated PCR can also be used to target conserved genes from other virus families. A general flowchart detailing the specific steps involved in the CODEHOP procedure to identify novel viral genes is shown in Figure 15. This procedure is based on the CODEHOP prediction software that we have previously developed and made accessible over the internet as part of the BLOCKS database [2]. An example of this procedure is provided below where CODEHOP PCR primers targeting the "DMGL" motif of herpesvirus TS genes (introduced above) are designed using the web-based software.

### Using the web-based software to design CODEHOP PCR primers to a conserved viral gene

**Figure 16:**
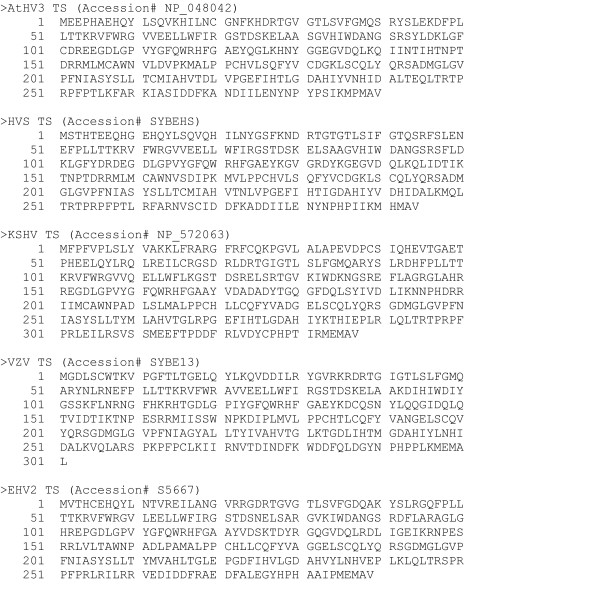
**Herpesvirus thymidylate synthase protein sequences**. The amino acid sequences of five herpesvirus TS genes used in the prediction of the DMGLXB and DMGLX1B CODEHOP PCR primers by the CODEHOP web-based software. The specific database accession numbers are indicated in the sequence title.

**Figure 17:**
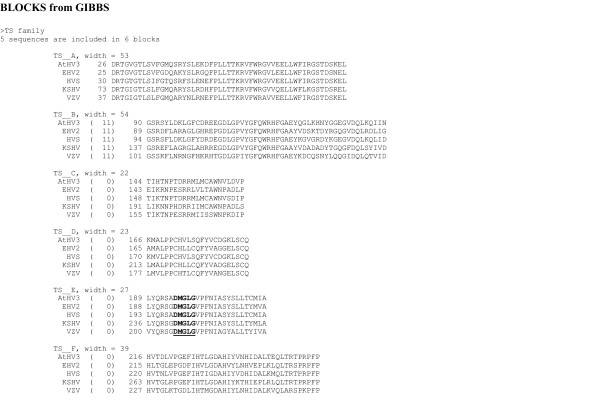
**Output of conserved sequence blocks obtained using the Gibbs method as implemented in the Block Maker program at the Blocks WWW server**. Six conserved sequence blocks were identified in the five herpesvirus TS genes shown in Figure 15. Block TS_E contains the DMGL motif (underlined) from which the DMGLXB and DMGLX1B complementary strand primers were derived.

**Figure 18:**
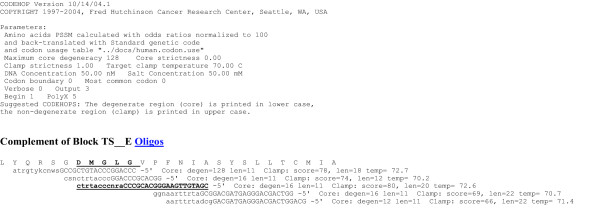
**Output of the web-based CODEHOP software predicting complementary strand CODEHOP PCR primers for the conserved "DMGL" motif of herpesvirus TS genes**. The TS_E block from the BlockMaker output in Figure 17 was provided as input to the CODEHOP software [3] and the PCR primers derived from the complementary strand are shown. The predicted consensus amino acid sequence is shown and the DMGL motif is underlined in bold. The complementary strand CODEHOP PCR primer selected for use in amplifying unknown TS genes is underlined in bold. The 3' degenerate core is shown in lowercase letters and the (len)gth and (degen)eracy are indicated. The 5' consensus clamp is shown in uppercase letters and the score, (len)gth and predicted melting (temp)erature are indicated.

The amino acid sequences of the TS genes from five herpesviruses were obtained using BLAST analysis of the NCBI protein database with the KSHV TS sequence as probe. The TS sequences from KSHV, VZV, EHV2, HVS and AtHV3 (Figure 16) were provided as input to ClustalW [28] and a multiple alignment was obtained. As shown in Figure 13, several regions of highly conserved sequences were present in the TS sequence alignment, and the positions of the "RHFG" and "DMGL" motifs targeted above are indicated. In order to predict CODEHOP PCR primers, the sequences of the TS genes were provided as input to the BlockMaker program of the Blocks Database [4] and a series of conserved sequence blocks were identified (ex., Gibbs Blocks, Figure 17). Alternatively, the ClustalW alignment, itself, could be provided as input to the "Multiple alignment processor" of the Blocks Database [29]. In order to compare a computer-predicted CODEHOP with the manually derived CODEHOP (DMGLB), the TS Block_E containing the "DMGL" motif (Figure 17) was directly input to the CODEHOP program [3] using all default values except that the consensus region was elongated by increasing the temperature setting from the default 60°C to 70°C. The primers predicted from the complement of Block_E were examined in order to obtain a primer from the complementary strand which could be used in conjunction with the upstream TS primer RHFGA, described above. The underlined primer targeting the "DMGL" motif was chosen and named DMGLXB (Figure 18) and was compared with the manually designed DMGLB primer in Figure 14. Whereas "DMGLB" was purposefully biased by using the KSHV sequences in the 5' consensus clamp, the "DMGLXB" is "unbiased" in design with the 5' consensus sequence derived from the most frequently used codons in the human genome. The DMGLXB sequence was examined for potential stem loop structures that could compromise the function of the primer. As shown in Figure 14, a putative stem-loop structure was identified which is indicated by the underlined nucleotides in Figure 14B and 14C. To destablize this structure, the proline codon within the "DMGLGVP" motif was changed from the computer predicted "CCC", the most frequently used codon in humans, to "CCA", another common human codon, as shown in Figure 14. This yielded a revised CODEHOP, called "DMGLX1B" (shown as the complementary sequence in Figures 14B and 14C), in which the stem-loop structure was destabilized by substituting an A for the highlighted C in Figure 14C. The DMGLX1B antisense primer could then be used in combination with the RHFGA sense primer to amplify unknown TS genes.

Other examples of CODEHOP PCR primers designed from multiple alignments of the herpesvirus DNA polymerase sequences using the Web-based CODEHOP software are shown in Figures 3, 4, 5, 6. The VYG1A primer designed from the conserved VYG motif shown in Figure 3 is aligned with the original manually designed "TGV" and "VYGA" primers. The computer-predicted "YGDTB" primer designed from the conserved GDTD motif is aligned with the original "IYG" and "GDTD1B" primers (Figure 4). In the prediction of this primer, the conserved sequence block identified by BlockMaker from the sequences shown in Figure 4A, extended only from amino acid position 1 – 10, which was the limit of the conserved sequence block determined by BlockMaker. The CODEHOP software indicated the necessity to add additional nucleotides to the 5' end of the "YGDTB" primer to obtain the minimal length for the 5' consensus region of the primer. As such, the amino acid sequences of block positions 11–13 were obtained manually and compared in order to derive the eight terminal nucleotides for "YGDTB" (overlined in Figure 4C).

## Conclusion

In this review, the utility of CODEHOP-mediated PCR for the identification of novel viruses and the characterization of new viral genes and genomic regions is presented. While the focus of this study was on the herpesvirus family, other virus families can be easily targeted using analogous approaches. We have previously developed successful CODEHOP assays targeting the reverse transcriptase genes of retroviruses and lentiviruses [2,6]. Recently, the CODEHOP strategy has been used to develop assays to detect novel papillomaviruses targeting the highly conserved L1 protein [30]. With the CODEHOP strategy, molecular sequence data can be readily obtained for comprehensive virus phylogenies and tracing of evolutionary pathways. Furthermore, comparison of multiple representatives of homologous viral proteins can be of importance for understanding the protein structure and function and provided insight into virus-host relationships.

## List of Abbreviations

CODEHOP, consensus-degenerate hybrid oligonucleotide primer; PCR, polymerase chain reaction; RFHV, retroperitoneal fibromatosis herpesvirus; KSHV, Kaposi's sarcoma-associated herpesvirus.

## Competing interests

The author(s) declare that they have no competing interests.

## Authors' contributions

Design, conception and preparation of the manuscript (TMR).
